# 163 Co-Production of a Physical Activity Intervention for Individuals with Severe Mental Illness in a Secure Forensic Ward – A Study Protocol

**DOI:** 10.1093/eurpub/ckae114.156

**Published:** 2024-09-26

**Authors:** Rebecca Feeney, Gavin Breslin, Martin Dempster, Stephen Shannon, Noel Brick, Gerard Leavey

**Affiliations:** Queens University Belfast, Belfast, United Kingdom; Queens University Belfast, Belfast, United Kingdom; Queens University Belfast, Belfast, United Kingdom; Ulster University, Coleraine, United Kingdom; Ulster University, Coleraine, United Kingdom; Ulster University, Coleraine, United Kingdom

## Abstract

**Background:**

Individuals living with Severe Mental Illness (SMI) experience significant health disparities in comparison to the general population including poorer physical health outcomes and reduced life expectancy. Physical activity (PA) plays a key role for improving physical and mental health outcomes in individuals living with SMI, especially those within secure forensic units. However, unique challenges exist in these settings, such as medication side effects, restrictive environments, lack of autonomy, and staff availability to accompany patient engagement in PA.

**Purpose:**

This study protocol describes a co-produce a physical activity intervention development for individuals living with SMI in a secure forensic ward. The intervention will incorporate psychological theory and linked behaviour change techniques, with the main objective of improving mental and physical health outcomes. The co-production model will firstly entail exploring barriers and facilitators influencing behavioural engagement in people with SMI. Second, understanding the staff’s role in promoting PA and establishing a theoretical framework to map PA behaviours throughout the intervention will be described.

**Method:**

The co-production process will involve patients with SMI, clinical staff, and university researchers. Theoretical Domains Framework (TDF) will be applied to identify implementation problems and determinants of behaviour, and will guide focus group discussions. The TIDieR checklist will be completed to provide a detailed intervention description.

**Results:**

The results will inform a co-produced physical activity intervention. The intervention will be embedded in behaviour change theory, incorporating behaviour change techniques.

**Conclusion:**

Understanding the factors that influence PA engagement in individuals living with SMI in secure forensic wards is crucial to developing effective interventions. Co- production of a theory-informed PA intervention will contribute to understanding methods to improve health outcomes in an understudied, hard-to-reach population residing in a challenging environment for achievement of behaviour change.

**Funding:**

This PhD programme of research is funded by the Department of Education (DfE).

